# A Rare Case of Dual De Novo Mutations Presenting With Infantile Spasms, Congenital Contractures, and Axial Hypotonia

**DOI:** 10.7759/cureus.98495

**Published:** 2025-12-04

**Authors:** Hadi Fakih

**Affiliations:** 1 Pediatrics, Faculty of Medical Sciences, Lebanese University, Beirut, LBN; 2 Neonatology, Sheikh Ragheb Harb University Hospital, Nabatieh, LBN

**Keywords:** cmpa, infantile epileptic spasm syndrome, joint contracture, neonatal hypotonia, whole exome sequencing (wes)

## Abstract

This report presents the case of a male infant with a complex neonatal course marked by congenital hand and foot deformities, axial hypotonia, and subsequent development of treatment-resistant infantile spasms. Despite extensive initial investigations yielding normal results, including a normal brain magnetic resonance imaging (MRI), whole exome sequencing (WES) revealed two distinct, likely de novo, autosomal dominant mutations: a pathogenic variant in the CACNA1E gene, consistent with Developmental and Epileptic Encephalopathy-69 (DEE69), and a likely pathogenic variant in the FBN1 gene, an incidental finding with significant implications for long-term cardiovascular health.

This case highlights the diagnostic power of WES in complex neurodevelopmental disorders and underscores the challenges in managing multifactorial presentations.

## Introduction

The diagnostic evaluation of a neonate or infant presenting with a constellation of neurological and skeletal abnormalities represents a significant challenge in pediatric neurology and genetics. When classic features, such as epileptic spasms, axial hypotonia, and congenital contractures, co-occur in the absence of structural brain anomalies on magnetic resonance imaging (MRI), the differential diagnosis narrows to specific genetic etiologies. Developmental and Epileptic Encephalopathies (DEEs) are a severe group of disorders characterized by early-onset, often refractory, seizures and developmental impairment or regression. DEE69 (Developmental and Epileptic Encephalopathy-69; OMIM #618285), caused by pathogenic variants in the CACNA1E gene, is one such disorder that frequently presents with this specific phenotypic combination, including arthrogryposis-like contractures [1,2].

The diagnostic odyssey in these cases is often prolonged, but the advent of comprehensive genetic testing, such as whole exome sequencing (WES), has revolutionized the ability to achieve a molecular diagnosis. Furthermore, WES can uncover unanticipated "secondary findings" - genetic variants unrelated to the primary clinical indication but with profound health implications, as outlined by the American College of Medical Genetics and Genomics (ACMG) [3]. This report details the complex case of an infant whose presentation of congenital contractures, infantile spasms, and hypotonia, despite a normal MRI, was ultimately explained by a de novo CACNA1E mutation, while also revealing an incidental FBN1 variant, illustrating the multifaceted impact of modern genetic diagnostics on patient management and family counseling.

## Case presentation

**Figure 1:**
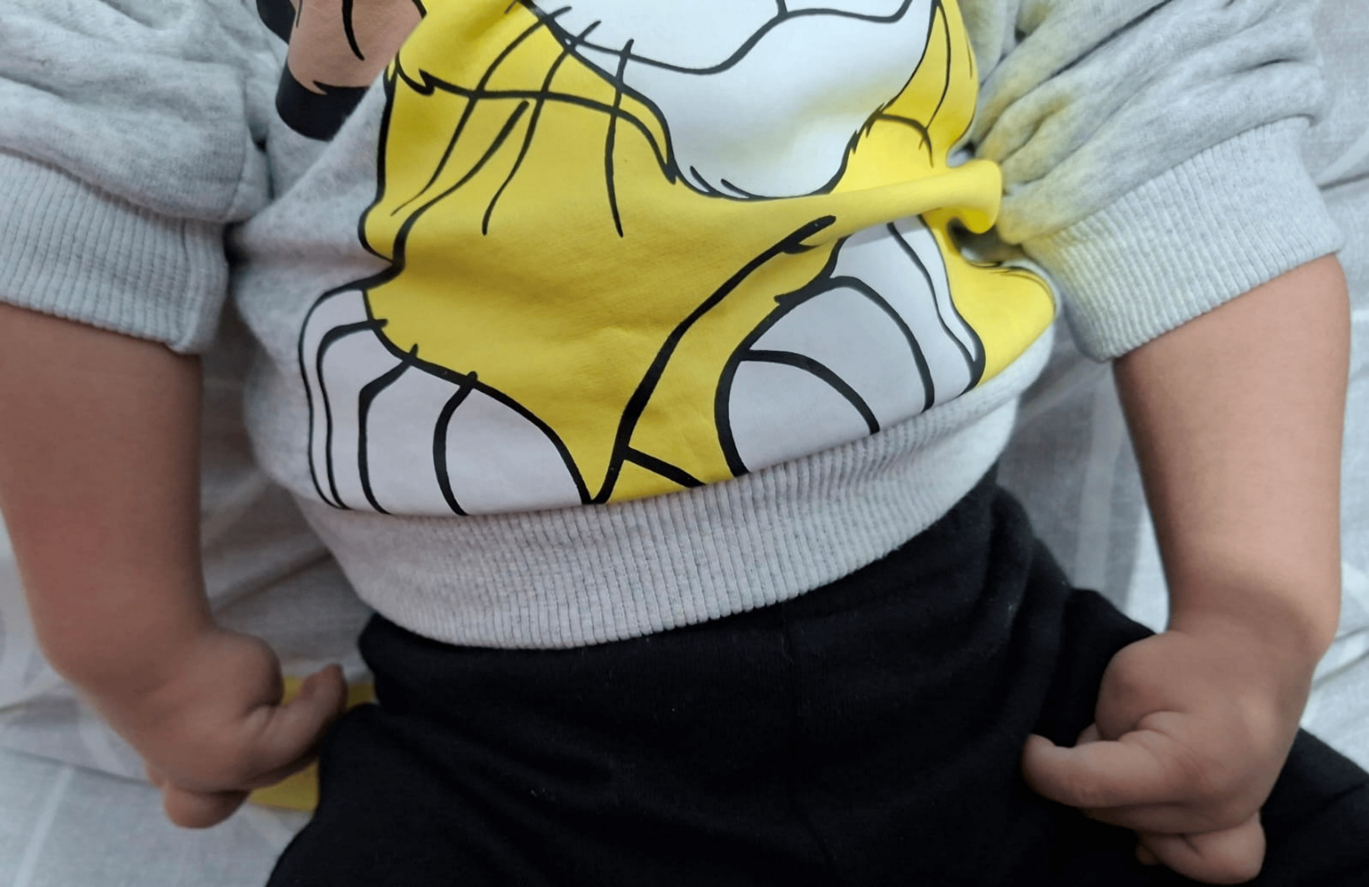
Bilateral clenched hands with spastic contractures, a characteristic feature often associated with DEE69 DEE69: Developmental and Epileptic Encephalopathy-69

**Figure 2:**
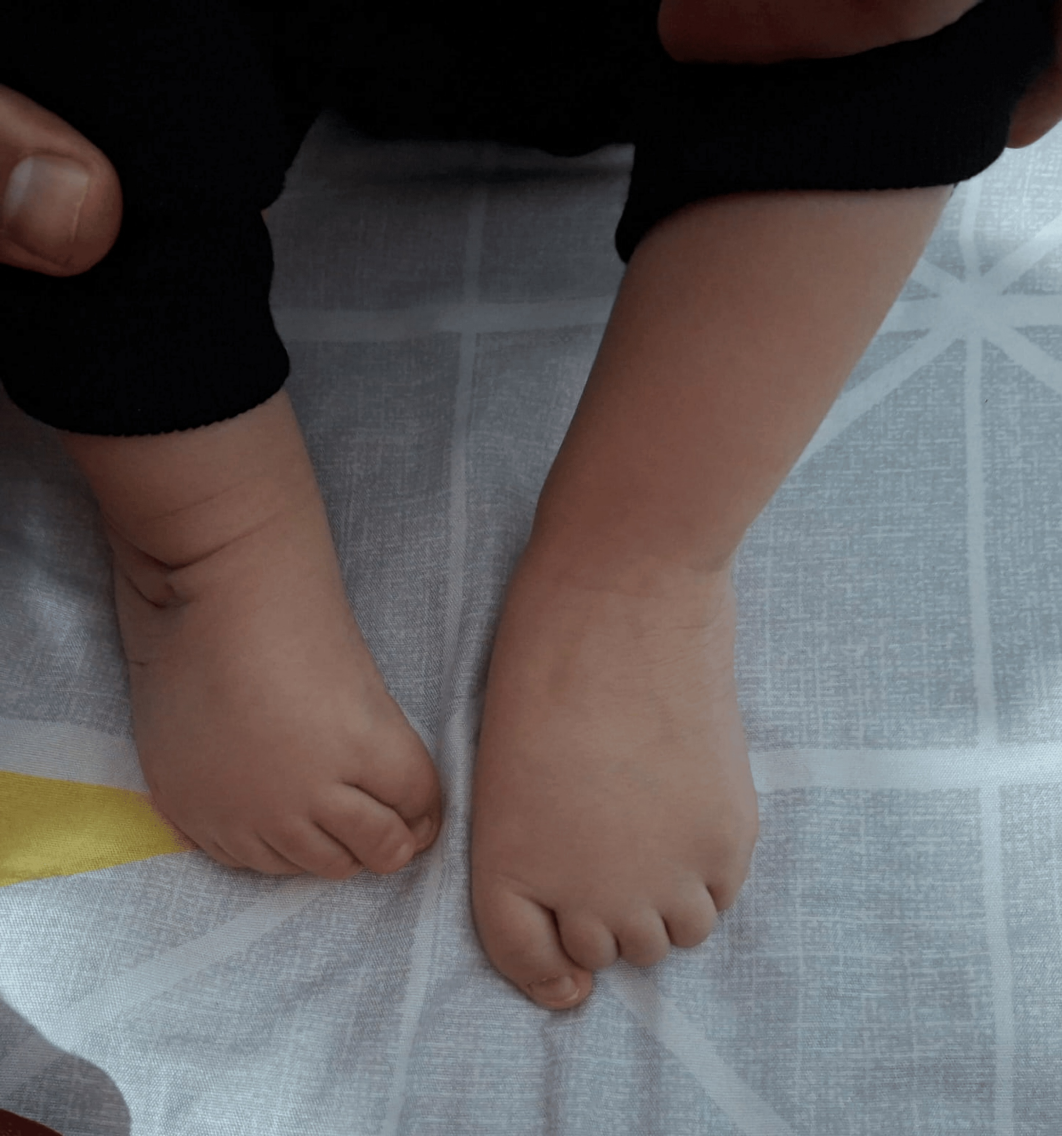
Bilateral club feet after serial casting procedures

A male newborn, born at full-term via programmed cesarean section to a 28-year-old mother (G5P1A4) with a history of recurrent abortions and a presumed maternal coagulation disorder, managed with subcutaneous enoxaparin during pregnancy. The pregnancy was otherwise unremarkable. There was a history of second-degree consanguinity on the maternal side. Birth weight was 2670g, and Apgar scores were 5 and 7 at 1 and 5 minutes, respectively, requiring positive pressure ventilation for less than one minute. The infant was admitted to the neonatal intensive care unit (NICU) for respiratory distress, which resolved with nasal continuous positive airway pressure (CPAP). Morphological examination revealed bilateral clenched hands with spastic contractures (Figure 1), bilateral club feet (Figure 2), and significant axial hypotonia. Initial investigations, including brain MRI, echocardiography, abdominal ultrasound, and metabolic screening, were all within normal limits. He was discharged home for ongoing skeletal physiotherapy.

Presentation at five months

The infant re-presented with continuous irritability, crying, refusal of oral intake, and poor weight gain. A trial of an amino acid formula and proton-pump inhibitors was initiated for suspected cow's milk protein allergy.

During this period, the mother provided a video of abnormal movements, highly suspicious for infantile spasms, which was subsequently confirmed by EEG. The patient was diagnosed with epileptic infantile spasms. Initial treatment with levetiracetam and clonazepam improved irritability but did not control the spasms. The regimen was escalated to include vigabatrin, and later, a third anti-epileptic drug, Vyncompa (perampanel), which successfully achieved seizure control. The constellation of skeletal deformities, infantile spasms, severe axial hypotonia, and a normal brain MRI prompted WES with copy number variation (CNV) analysis.

Primary finding (CACNA1E)

A heterozygous, de novo pathogenic variant (c.2104G>A, p.Ala702Thr) was identified. This is causative for DEE69, an autosomal dominant disorder that perfectly explains the patient's core neurological and skeletal phenotype.

Secondary/incidental finding (FBN1)

A heterozygous, likely pathogenic frameshift variant (c.796delT, p․Ser268LeufsTer62) was identified. This gene is associated with Marfan syndrome and aortopathies, necessitating lifelong cardiovascular surveillance.

## Discussion

This case offers several critical learning points for the management of complex infantile neurological disorders. A normal brain MRI in an infant with infantile spasms, hypotonia, and congenital deformities often redirects the diagnostic focus from structural anomalies to metabolic or genetic etiologies. While the metabolic workup was negative, WES provided a definitive diagnosis. DEE69, caused by gain-of-function mutations in the CACNA1E-encoded voltage-gated calcium channel CaV2.3, is characterized by early-onset refractory seizures, profound developmental delay, movement disorders, congenital contractures (as seen in this patient), and visual abnormalities [1]. The presence of congenital contractures should heighten suspicion for specific genetic encephalopathies like DEE69, even in the absence of MRI findings [2].

This case also illustrates a typical stepwise approach to refractory epilepsy management. After first-line treatments for infantile spasms (clonazepam and vigabatrin) are tried, combination therapy with multiple anti-seizure medications (ASMs) is often necessary [4]. The successful control with a combination of vigabatrin, perampanel, and clonazepam underscores the need for persistent, tailored therapeutic strategies in DEEs. The use of perampanel, an AMPA receptor antagonist, highlights the evolving approach of targeting specific neurotransmitter systems in refractory cases [5].

The identification of a de novo, pathogenic CACNA1E variant carries significant prognostic implications. DEE69 is associated with severe to profound intellectual disability and motor impairment. Most children are non-ambulatory and non-verbal, and seizures may remain refractory in some cases [1,6]. Early genetic diagnosis allows for the cessation of the diagnostic odyssey, informed prognostic counseling for the family, and personalized management plans focusing on anticipatory guidance and targeted rehabilitation [7].

The finding of de novo mutations is crucial for genetic counseling. A de novo event means the variant arose spontaneously in the proband and was not inherited from either parent. Consequently, the recurrence risk for parents to have another child with the same disorder is very low (<1%) [8]. This information is profoundly important for family planning, particularly in this case with a history of recurrent abortions, as it effectively decouples the child's genetic condition from the mother's reproductive history. However, germline mosaicism cannot be entirely ruled out, making prenatal diagnosis a recommended option for future pregnancies [8].

The challenge of incidental findings by the discovery of a likely pathogenic FBN1 variant introduces a separate, lifelong healthcare mandate. While unrelated to the neurological presentation, it necessitates regular cardiovascular evaluations to monitor for aortic root dilation and risk of dissection, in line with the management guidelines for Marfan syndrome and related aortopathies [9]. Genetic counseling for the proband's parents and potentially other first-degree relatives for cascade testing is imperative, as recommended by the American College of Medical Genetics and Genomics (ACMG) for secondary findings [3].

## Conclusions

This case exemplifies the paradigm of precision medicine in modern neurology. A child with a seemingly inexplicable combination of severe neurological, skeletal, and gastrointestinal symptoms was found to have a dual genetic diagnosis: a de novo CACNA1E mutation causing DEE69 and an incidental FBN1 finding. The WES result provided a definitive explanation for the infantile spasms and contractures, offered a clear prognosis, guided therapeutic decisions, and provided critical reassurance regarding recurrence risk for the family. Furthermore, it unveiled an unrelated but serious health risk, enabling pre-symptomatic surveillance and preventive care. This report underscores the indispensable role of comprehensive genetic testing in unraveling complex pediatric neurodevelopmental disorders.

## References

[1] Helbig KL, Lauerer RJ, Bahr JC (2018). De novo pathogenic variants in CACNA1E cause developmental and epileptic encephalopathy with contractures, macrocephaly, and dyskinesias. Am J Hum Genet.

[2] Howell KB, Eggers S, Dalziel K (2018). A population-based cost-effectiveness study of early genetic testing in severe epilepsies of infancy. Epilepsia.

[3] Go CY, Mackay MT, Weiss SK, Stephens D, Adams-Webber T, Ashwal S, Snead OC 3rd (2012). Evidence-based guideline update: medical treatment of infantile spasms [RETIRED]. Report of the Guideline Development Subcommittee of the American Academy of Neurology and the Practice Committee of the Child Neurology Society. Neurology.

[4] French JA, Krauss GL, Biton V (2012). Adjunctive perampanel for refractory partial-onset seizures: randomized phase III study 304. Neurology.

[5] Heyne HO, Singh T, Stamberger H (2018). De novo variants in neurodevelopmental disorders with epilepsy. Nat Genet.

[6] Srivastava S, Love-Nichols JA, Dies KA (2019). Meta-analysis and multidisciplinary consensus statement: exome sequencing is a first-tier clinical diagnostic test for individuals with neurodevelopmental disorders. Genet Med.

[7] Campbell IM, Shaw CA, Stankiewicz P, Lupski JR (2015). Somatic mosaicism: implications for disease and transmission genetics. Trends Genet.

[8] Hiratzka LF, Bakris GL, Beckman JA (2010). 2010 ACCF/AHA/AATS/ACR/ASA/SCA/SCAI/SIR/STS/SVM guidelines for the diagnosis and management of patients with thoracic aortic disease: a report of the American College of Cardiology Foundation/American Heart Association Task Force on Practice Guidelines, American Association for Thoracic Surgery, American College of Radiology, American Stroke Association, Society of Cardiovascular Anesthesiologists, Society for Cardiovascular Angiography and Interventions, Society of Interventional Radiology, Society of Thoracic Surgeons, and Society for Vascular Medicine. Circulation.

[9] Kalia SS, Adelman K, Bale SJ (2017). Recommendations for reporting of secondary findings in clinical exome and genome sequencing, 2016 update (ACMG SF v2.0): a policy statement of the American College of Medical Genetics and Genomics. Genet Med.

